# Plant protein glycosylation

**DOI:** 10.1093/glycob/cww023

**Published:** 2016-10-01

**Authors:** Richard Strasser

**Affiliations:** 2 Department of Applied Genetics and Cell Biology, University of Natural Resources and Life Sciences, Vienna, Muthgasse 18, 1190 Vienna, Austria

**Keywords:** endoplasmic reticulum, glycosyltransferase, Golgi apparatus, *N*-glycan processing, N-glycosylation

## Abstract

Protein glycosylation is an essential co- and post-translational modification of secretory and membrane proteins in all eukaryotes. The initial steps of N-glycosylation and *N*-glycan processing are highly conserved between plants, mammals and yeast. In contrast, late *N*-glycan maturation steps in the Golgi differ significantly in plants giving rise to complex *N*-glycans with β1,2-linked xylose, core α1,3-linked fucose and Lewis A-type structures. While the essential role of *N*-glycan modifications on distinct mammalian glycoproteins is already well documented, we have only begun to decipher the biological function of this ubiquitous protein modification in different plant species. In this review, I focus on the biosynthesis and function of different protein N-linked glycans in plants. Special emphasis is given on glycan-mediated quality control processes in the ER and on the biological role of characteristic complex *N*-glycan structures.

## Introduction

**Fig. 1.**
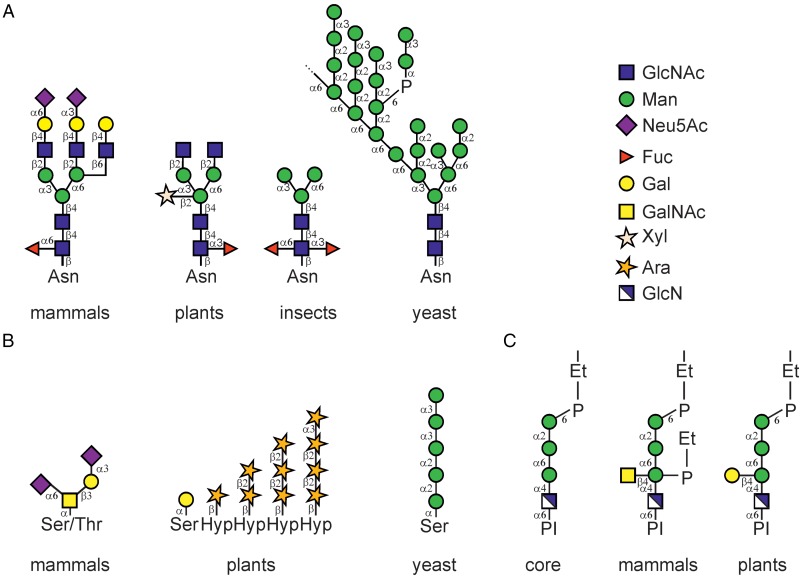
Comparison of different types of protein linked glycans. (**A**) Typical *N*-glycan structures from mammals, plants, insects and yeast (*S. cerevisiae*) are shown. The symbols for the monosaccharides in the illustration are drawn according to the nomenclature from the Consortium for Functional Glycomics. (**B**) Schematic representation of characteristic *O*-glycans from mammals (di-sialylated core 1), plants (extensin-type modification on Ser and contiguous hydroxyproline (Hyp) residues, the structure is drawn according to Nguema-Ona et al. 2014) and *S. cerevisiae*. The carbohydrate structure of an *A. thaliana* arabinogalactan-protein is not shown here and can be found in Tryfona et al. (2012). (**C**) The conserved core glycan structure of the GPI-anchor is shown as well as examples derived from a human GPI-anchored protein (Kinoshita 2014) and from pear cells (Oxley and Bacic 1999). P-Et indicates the phsphoethanolamine linkage. This figure is available in black and white in print and in color at *Glycobiology* online.

Asparagine (N)-linked glycosylation (ALG) of proteins is the most common co- and post-translational modification of proteins entering the secretory pathway. N-Glycosylation plays an important role for many biological processes including protein folding, glycan-dependent quality control processes in the ER, protein stability and protein–protein interactions (Moremen et al. 2012; Hebert et al. 2014). To date, more than thousand different N-glycosylated proteins have been identified with high confidence in the model plant *Arabidopsis thaliana* (Zielinska et al. 2012; Song et al. 2013). These proteins have a confirmed or predicted location in the secretory pathway and carry one or several *N*-glycans. The number of glycoproteins and identified glycosylation sites appear similar to animal model species like *Drosophila melanogaster* and *Danio rerio* (Zielinska et al. 2012). In contrast to mammals, however, plants produce oligosaccharides of reduced complexity and diversity as they lack, for example, branched and sialylated *N*-glycans (Figure 1A). Based on findings from *A. thaliana* (von Schaewen et al. 1993; Strasser et al. 2004), it has been hypothesized that complex *N*-glycans are not essential for the development and reproduction of plants when grown under standard environmental conditions. Strikingly, however, all of the characteristic complex *N*-glycan modifications (β1,2-Xyl, core α1,3-Fuc and Lewis A-type structures) are conserved in higher plants and even found in distantly related mosses like *Physcomitrella patens* (Fitchette et al. 1999; Wilson, Zeleny, et al. 2001; Viëtor et al. 2003), suggesting that there are evolutionary constraints that prevent the loss of these *N*-glycan modification. For a long time, it remained obscure why plants produce distinct complex *N*-glycans at all and whether there is any specific function related to complex *N*-glycan modifications (Lerouge et al. 1998). This review focuses on recent findings concerning the biological role of oligomannosidic and complex *N*-glycans in different plants.

Apart from N-glycosylation other types of protein glycosylation have been described in plants. Similar to mammals, the transfer of a single GlcNAc to Ser/Thr residues (O-GlcNAcylation) of cytosolic or nuclear proteins appears to play an important role in cellular signaling (Olszewski et al. 2010). O-Glycosylation, another major type of protein glycosylation, is fundamentally different in plants (Figure 1B). Mucin-type *O*-glycans have not been detected on native plant proteins and the glycosyltransferases for initiation and elongation of mucin-type *O*-glycans have not been found in the plant genomes. In plants, on the other hand, a single Gal can be transferred to Ser residues on specific proteins and arabinose chains as well as structurally complex arabinogalactans occur on hydroxyproline residues of cell wall proteins (Taylor et al. 2012; Tryfona et al. 2012; Saito et al. 2014). A bioinformatics approach has identified 166 hydroxyproline-rich glycoproteins including 85 putative arabinogalactan proteins (Showalter et al. 2010). Aspects of their biosynthesis and functions have been summarized in recent reviews and are not further addressed here (Tan et al. 2012; Hijazi et al. 2014; Nguema-Ona et al. 2014). In yeast, O-mannosylation in the endoplasmic reticulum (ER) plays an important role for protein folding and quality control (Xu et al. 2013). This and other types of *O*-glycan modifications have also not been detected on plant secretory or membrane proteins. However, the recent identification of the ER-resident nucleotide sugar transporter ROCK1 in *A. thaliana* hints that an unknown protein glycosylation modification might exist in plants with a similar role in protein quality control (Niemann et al. 2015). Using transport assays in yeast, it has been shown that ROCK1 transports mainly the nucleotide sugars UDP-GalNAc and UDP-GlcNAc. In *A. thaliana* this transport activity is apparently involved in the regulation of the plant response to the hormone cytokinin. How this is achieved and whether it requires an as yet undiscovered protein glycosylation in the ER of plants remains to be shown in the future.

The attachment of a glycosylphoshatidylinositol (GPI) anchor to the C-terminus of proteins is another common protein modification in the ER of plants. A combination of proteome analysis and bioinformatic search in the *A. thaliana* genome identified 248 putative GPI-anchored proteins (Borner et al. 2003). The GPI-anchor is synthesized by a stepwise process involving a conserved protein machinery and transferred *en bloc* to the protein by the GPI-transamidase complex (Kinoshita 2014). The core glycan structure found in all eukaryotic GPI-anchors comprises Manα(1–2)Manα(1–6)Manα(1–4)GlcN-inositol (Figure 1C). In many eukaryotes, the core glycan can be further modified by incorporation of different sugars including Man and GalNAc. The glycan composition of only one GPI-anchor has been determined in plants to date. The GPI-anchored arabinogalactan-protein derived from a *Pyrus communis* cell suspension culture carried the conserved core oligosaccharide that was partially modified with a β1–4Gal residue (Oxley and Bacic 1999). Whether the glycan moiety of plant GPI-anchored proteins displays species-, cell-type- and protein-specific variations remain to be shown in the future. Putative plant orthologs of the different GPI biosynthesis proteins have been identified in *A. thaliana* and rice (Eisenhaber et al. 2003; Ellis et al. 2010), but most of them have not been functionally characterized. Heterozygous mutants lacking the putative *A. thaliana* GPI-GlcNAc transferases (SETH1: a homolog of mammalian PIG-C and SETH2: a homolog of mammalian PIG-A) showed male-specific defects in fertility (Lalanne et al. 2004). Likewise, plants with a disrupted homolog of the mammalian α1,4-mannosyltransferase PIG-M (termed PEANUT1) displayed reduced amounts of GPI-anchored proteins and were seedling lethal (Gillmor et al. 2005). In a recent study, a mutation in the *A. thaliana* homolog of the α1,2-mannosyltransferase PIG-B (termed APTG1) was found to display similar phenotypes with abnormal male fertility and embryo lethality (Dai et al. 2014). Together, these studies highlight that correct GPI-core oligosaccharide assembly is essential for the vegetative and reproductive development of plants.

## Assembly of lipid-linked *N*-glycans in plants

**Fig. 2.**
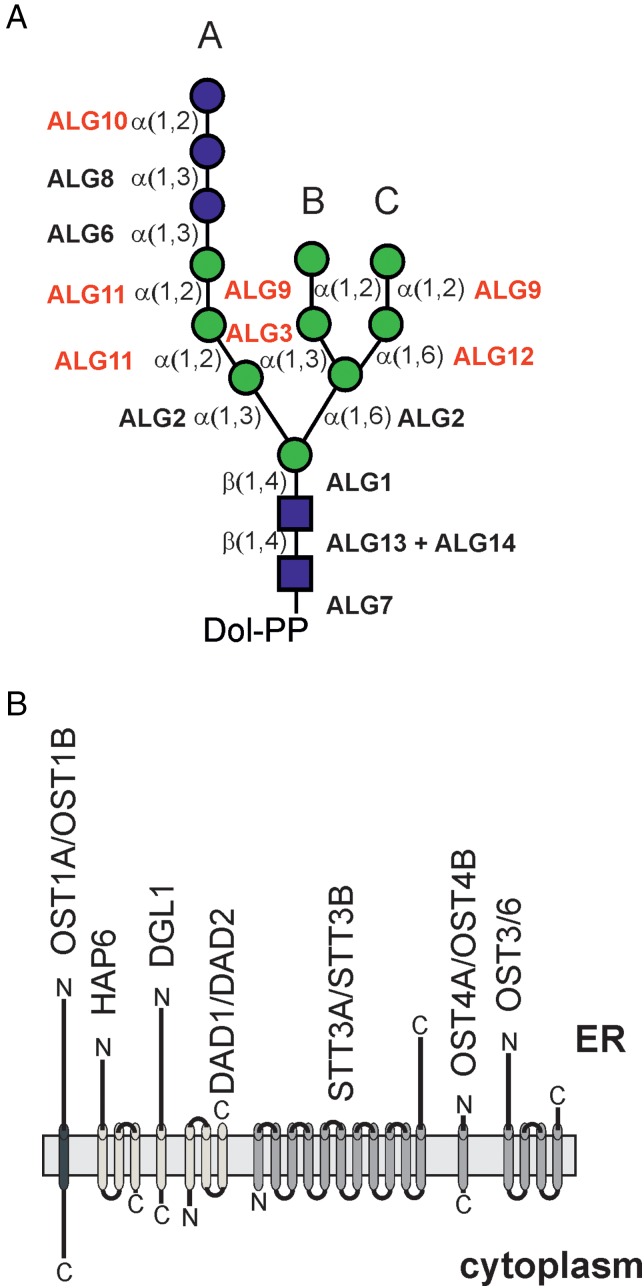
(**A**) ALG proteins involved in lipid-linked oligosaccharide precursor biosynthesis. Genetic evidence for their function has been shown for the *A. thaliana* glycosyltransferases highlighted in a different color. (A), (B) and (C) define the different branches of the oligosaccharide. (**B**) Illustration of the putative plant OST complex consisting of different subunits. The proposed topology of the enzymes is depicted. Please note that for some proteins like STT3A/STT3B the exact number of transmembrane domains is unclear (Koiwa et al. 2003) and may range from 10 to 14 helices. The different colors of the transmembrane domains denote subcomplexes that have been described for the yeast and mammalian OST complex (Kelleher and Gilmore 2006). This figure is available in black and white in print and in color at *Glycobiology* online.

In all eukaryotes, a hallmark of N-glycosylation is the *en bloc* transfer of the pre-assembled Glc_3_Man_9_GlcNAc_2_ oligosaccharide from the lipid carrier dolichol pyrophosphate to selected asparagine residues in the sequence Asn-X-Ser/Thr (X can be any amino acid except proline) within nascent polypeptides. The enzymatic steps leading to the biosynthesis of the lipid-linked precursor appear the same in plants (Figure 2A). All known yeast ALG proteins are encoded by plant genomes (Lannoo and Van Damme 2015) and *A. thaliana* mutants with defects in different steps of the lipid-linked oligosaccharide assembly pathway have been described. Loss of ALG3 (Henquet et al. 2008; Kajiura, Seki, et al. 2010), ALG12 (Hong et al. 2009) and ALG9 (Hong et al. 2012) function is well tolerated by the plants and does not cause any growth or developmental phenotype. In contrast, ALG11 null mutants are lethal (Zhang et al. 2009) and plants with ALG10 deficiency display reduced glycosylation efficiency and a leaf growth defect (Farid et al. 2011). The topology of the ALG proteins and their site of action (cytosolic side or ER-lumen) have not been studied in detail in plants. Based on the overall conservation of the pathway, it is expected that ALG11 is active at the cytosolic side of the ER and transfers two consecutive α1,2-linked Man residues to the lipid-linked oligosaccharide. The resulting Man_5_GlcNAc_2_-PP-Dol is then very likely transported across the ER membrane by a flippase-like protein and used as substrate in the ER lumen by ALG3, ALG9, ALG12 and the three glucosyltransferases (ALG6, ALG8 and ALG10).

## The plant oligosaccharyltransferase complex

The *en bloc* transfer of the pre-assembled oligosaccharide to an asparagine residue in the canonical N-glycosylation acceptor site takes place in the lumen of the ER and is catalyzed by the oligosaccharyltransferase (OST) complex. The use of alternative N-glycosylation sites like Asn-X-Cys is possible in plant cells (Matsui et al. 2011), but has not been described for endogenous plant proteins (Zielinska et al. 2010). In yeast and mammals, OST is a heteromeric membrane-bound protein complex consisting of one catalytically active subunit (staurosporine and temperature sensitivity 3, STT3) and several different non-catalytic subunits that modulate N-glycosylation by regulation of the substrate specificity, stability or assembly of the complex (Kelleher and Gilmore 2006; Mohorko et al. 2011). The yeast OST complex is composed of a single STT3 protein and seven additional subunits (Kelleher and Gilmore 2006). In metazoans, the organization of the OST complex appears more complex and different subunit compositions have been proposed (Shibatani et al. 2005; Mohorko et al. 2011; Roboti and High 2012). Notably, mammals harbor two different catalytic isoforms (STT3A and STT3B) that are present in distinct OST complexes (Ruiz-Canada et al. 2009; Shrimal and Gilmore 2013; Shrimal et al. 2015). The mammalian STT3A- and STT3B-containing complexes have overlapping as well as isoform-specific functions and differ in their acceptor substrate selectivity. While human STT3A is predominately involved in co-translational glycosylation, STT3B displays a preference for post-translational glycosylation.

The transfer of the pre-assembled oligosaccharide in plants involves a similar OST multi-subunit complex (Figure 2B), which is still poorly characterized. In *A. thaliana* two proteins, termed STT3A and STT3B, with homology to the yeast and mammalian catalytic subunits have been identified (Koiwa et al. 2003) (Table I). STT3A-deficient plants are viable, but display a protein underglycosylation defect that affects the biogenesis of heavily glycosylated proteins, such as the pattern recognition receptor EF-TU RECEPTOR (EFR) (Nekrasov et al. 2009; Saijo et al. 2009; Häweker et al. 2010). In contrast, STT3B-deficiency does not lead to any obvious changes in N-glycosylation efficiency and EFR function is not compromised (Koiwa et al. 2003; Nekrasov et al. 2009; Häweker et al. 2010). Even though, the *A. thaliana* STT3B subunit is more closely related to human STT3A than to human STT3B, no substrate has yet been identified for STT3B. However, *A. thaliana stt3a stt3b* double knockout plants are gametophytic lethal (Koiwa et al. 2003). These data highlight the importance of the catalytic OST subunits for N-glycosylation of plant proteins and reveal that the two putative catalytic subunits have overlapping as well as substrate-specific functions. Homologs of the two different *A. thaliana* STT3 subunits are also found in many other plant species, indicating that plants have also two functionally distinct OST complexes.

**Table I.** Putative subunits of the *A. thaliana* OST complex

*A. thaliana* OST subunit	Locus	*S. cerevisiae* homolog	Amino acid identity in % to the yeast subunit	Reference
DGL1	At5g66680	Wbp1p	25	Lerouxel et al. (2005)
STT3A STT3B	At5g19690 At1g34130	Stt3p Stt3p	47 49	Koiwa et al. (2003) Koiwa et al. (2003)
OST1A OST1B	At2g01720 At1g76400	Ost1p Ost1p	23 24	
HAP6	At4g21150	Swp1p	25	Johnson et al. (2004)
DAD1 DAD2	At1g32210 At2g35520	Ost2p Ost2p	36 36	Gallois et al. (1997) Danon et al. (2004)
OST4A OST4B	At3g12587 At5g02502	Ost4p Ost4p	31 26	Farid et al. (2013)
OST3/6 OST3/6-LIKE1	At1g61790 At1g11560	Ost3p/Ost6p Ost3p/Ost6p	<17 <17	Farid et al. (2013) Farid et al. (2013)

No homolog of the yeast Ost5p subunit has been identified in the *A. thaliana* genome. The amino acid identity between DAD1 and DAD2 is ∼95% and OST4A/OST4B share ∼88% identity. In contrast, the amino acid identity of OST1A and OST1B to each other is <50%, suggesting that they represent two OST1 isoforms with little or only partially overlapping function.

In another study, it has been shown that depletion of *A. thaliana* defective glycosylation 1 (DGL1), a homolog of the essential yeast subunit wheat germ agglutinin binding protein 1 (Wbp1p), is embryo lethal (Lerouxel et al. 2005). Consistent with a role in N-glycosylation, an *A. thaliana* mutant with a weak *dgl1* allele displays reduced protein N-glycosylation occupancy and a partial loss-of-function mutation in the gene coding for rice DGL1 causes defects in root formation (Lerouxel et al. 2005; Qin et al. 2013). The *A. thaliana* homolog (OST3/6) of yeast Ost3p/Ost6p is required for efficient N-glycosylation of EFR (Farid et al. 2013) and together with STT3A OST3/6 is involved in a specific plant cell death response (de Oliveira et al. 2016). Yet, the precise molecular function of OST3/6 and the role of the related OST3/6-LIKE1 in N-glycosylation of plants remain to be established. Whether these proteins either have an oxidoreductase activity or bind to the polypeptide substrates like it has been proposed for the yeast proteins is unclear (Schulz et al. 2009; Jamaluddin et al. 2014). A preliminary biochemical characterization suggests that OST3/6, STT3A and OST4B, a homologue of the yeast Ost4p subunit, interact *in planta* and form an OST sub-complex similar to that of *Saccharomyces cerevisiae* (Kim et al. 2003; Farid et al. 2013).

Additional *A. thaliana* proteins like hapless 6 (HAP6) and defender against apoptotic cell death 1/2 (DAD1/DAD2) have been identified (Table I), which are putative orthologs of yeast suppressor of Wbp1 (Swp1p)/mammalian ribophorin II and OST2/DAD1, respectively (Gallois et al. 1997; Danon et al. 2004; Johnson et al. 2004). *Arabidopsis thaliana* DAD1 can rescue the apoptotic phenotype of a mutant hamster cell line indicating a conserved function between animals and plants (Gallois et al. 1997). However, a specific role in N-glycosylation has yet to be revealed for DAD1/DAD2 and HAP6. Two putative orthologs of mammalian ribophorin I and yeast Ost1p which share <50% amino acid identity to each other are present in *A. thaliana* but these OST1 candidates have not been functionally characterized yet and their involvement in N-glycosylation remains to be examined. Amino acid sequence comparison between *A. thaliana* proteins and *S. cerevisiae* subunits reveals that the catalytic STT3A/STT3B subunits are better conserved in plants than all the other putative subunits (Table I). Nonetheless, a prediction of the protein topologies suggests that they are highly similar between species (Figure 2B). Based on these data, it is therefore possible that some OST subunits have a conserved role in the complex while others might have a different function in plants. Further insights into OST function await the purification of the complex, identification of the subunit composition and subsequent biochemical characterization.

## *N*-Glycan processing in the ER of plants

**Fig. 3.**
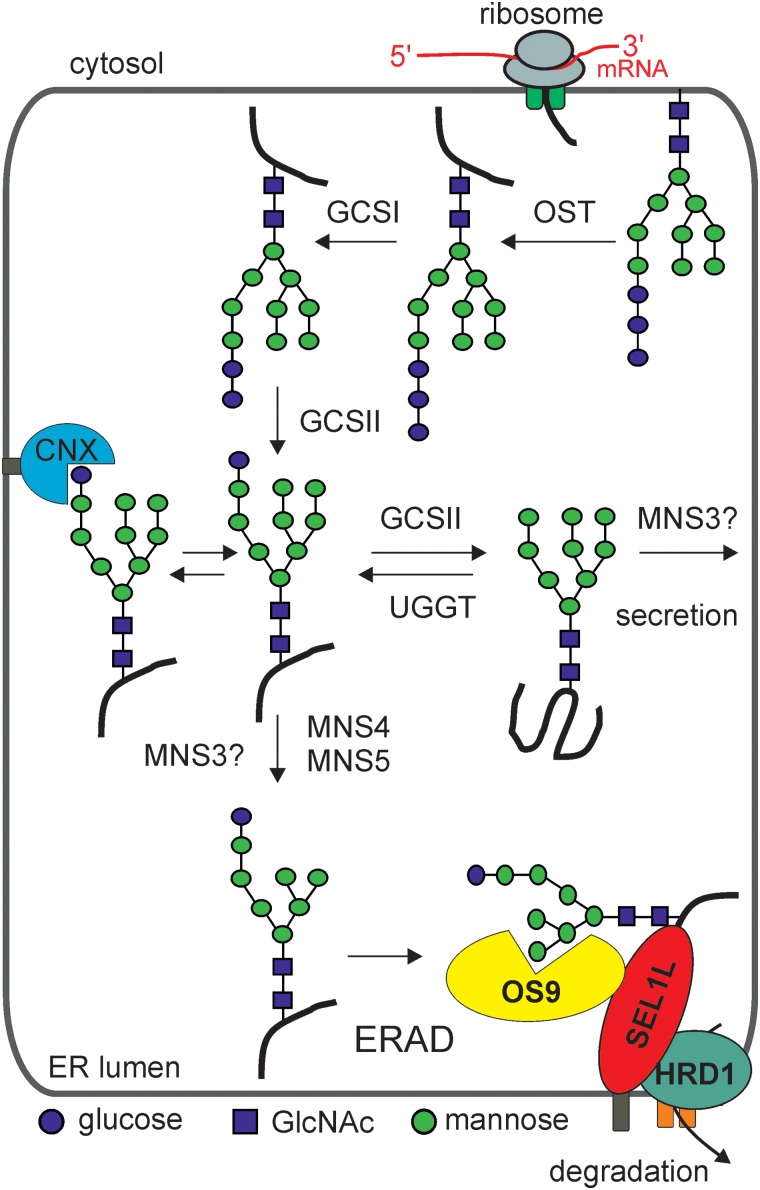
*N*-Glycan processing and *N*-glycan-mediated quality control in the ER of plants. The OST complex transfers the assembled *N*-glycan precursor to accessible Asn residues within the glycosylation consensus sites of nascent polypeptides. The first *N*-glycan processing step is carried out by α-glucosidase I (GCSI). Upon trimming of another terminal Glc residue by GCSII, the protein with a mono-glucosylated *N*-glycan may enter the calnexin (CNX)/calreticulin cycle. Proper folded glycoproteins are released from the quality control process and can exit the ER. Aberrant glycoproteins that cannot attain their final conformation are sent for degradation by the ERAD pathway which requires MNS4/MNS5-mediated Man trimming and recognition by OS9. The class I α-mannosidase MNS3 hydrolyses a single α1,2-Man residue from the middle branch (B-branch see also Figure 2A) of the oligomannosidic *N*-glycan. MNS3 may act on folded as well as on partially folded glycoproteins. The subcellular site of MNS3 action is still obscure. While MNS3 has so far been exclusively found in Golgi-like structures (Liebminger et al. 2009), ER-resident glycoproteins display *N*-glycans that have been trimmed by the MNS3-like ER-α-mannosidase activity. This figure is available in black and white in print and in color at *Glycobiology* online.

Upon transfer of the Glc_3_Man_9_GlcNAc_2_ oligosaccharide to the asparagine residue of a nascent polypeptide chain, the *N*-glycan is processed in the lumen of the ER. Like in other eukaryotes, the first trimming reactions are performed by α-glucosidases I (GCSI) and α-glucosidases II (GCSII) which remove the outermost two Glc residues. Before being subjected to further processing, incompletely folded glycoproteins with mono-glucosylated glycans can be captured by the ER resident lectins calnexin (CNX) and calreticulin. As part of a conserved quality control process, this specific lectin–glycan interaction prevents aggregation of folding intermediates and promotes folding of glycoproteins (Caramelo and Parodi 2008). In mammals, Glc trimming by GCSII releases the protein from CNX/calreticulin, while re-glucosylation by the folding sensor UDP-Glc glycoprotein glucosyltransferase (UGGT) enables again the interaction with CNX/calreticulin. Glycoproteins that attain their final conformation are no longer recognized by UGGT and allowed to exit the ER to downstream compartments (Figure 3).

GCSI activity is essential for plants and mutants with weak *gcsI* alleles display severe growth defects (Boisson et al. 2001; Gillmor et al. 2002; Furumizu and Komeda 2008; Wang et al. 2014) (Table II). However, these defects can be suppressed by additional knockout of the α1,2-glucosyltransferase ALG10 (Farid et al. 2011), indicating that trimming of the outermost Glc is a prerequisite for further processing by GCSII. Plant GCSII occurs very likely as a luminal hetero-dimeric protein consisting of the catalytic α-subunit (GCSIIα) and the Man-6-phosphate receptor homology domain-containing β-subunit (GCSIIβ) (Kaushal et al. 1990; Soussilane et al. 2009). Despite several attempts, *gcsIIα* null mutants could not be obtained so far, suggesting that the GCSIIα-mediated Glc trimming reactions are essential for plants (Burn et al. 2002; Soussilane et al. 2009). Weaker alleles of *gcsIIα* or plants with reduced GCSIIα expression are viable, but display severe developmental defects (Taylor et al. 2000; Burn et al. 2002). In contrast, GCSIIβ-deficient plants lack any phenotype under normal growth conditions. Nonetheless, GCSIIβ is required for proper EFR biogenesis and *gcsIIβ* plants display changes in defense signaling and plant immunity (Lu et al. 2009; von Numers et al. 2010). Given the central role of UGGT in ER quality control processes in mammals (Molinari et al. 2005), it is surprising that *A. thaliana* plants deficient for the single UGGT isoform are viable and do not display any obvious growth or developmental phenotype (Jin et al. 2007). In view of the essential role of GCSII, it is possible that a single CNX/calreticulin interaction is sufficient to achieve proper folding of the important plant glycoproteins. However, another study suggests that *A. thaliana* with either no or low UGGT expression are impaired in their growth and more sensitive towards biotic and abiotic stress treatments which contradicts the previous findings (Blanco-Herrera et al. 2015). In addition, different genetic screens have identified *A. thaliana* lines with mutations in the gene coding for UGGT (Jin et al. 2007; Li et al. 2009; Saijo et al. 2009; Zhang et al. 2015). These studies demonstrate that UGGT is critical for the biogenesis of a mutant variant of BRASSINOSTEROID INSENSITIVE1 (BRI1-9), EFR and SUPPRESSOR OF BIR1,1 (SOBIR1), which are all glycosylated receptor-like kinases. Further studies are needed to solve the reported discrepancy of UGGT function and to better understand its role for glycan-mediated quality control.

**Table II.** Overview of plant *N*-glycan processing mutants and their phenotypes

Protein	Locus	Mutant	Phenotype	Reference
*A. thaliana* GCSI (MOGS)^a^	At1g67490	*gcsI* *knf14* *knf101*	Embryo lethal Embryo lethal Hypomorphic allele, growth phenotype	Boisson et al. (2001) Gillmor et al. (2002) Furumizu and Komeda (2008)
GCSII α (GANAB)	At5g63840	*rsw3* *psl5-1*	Hypomorphic allele, temperature-sensitive root growth phenotype Hypomorphic allele, defect in EFR biogenesis, altered plant immunity	Burn et al. (2002) Lu et al. (2009)
GCSII β (GLU2B)	At5g56360	*gcsIIβ* *psl4-1* *psl4-2*	Impaired plant immunity against bacteria, defect in EFR biogenesis, altered plant immunity	von Numers et al. (2010) Lu et al. (2009)
MNS3 (MAN1B1)	At1g30000	*mns3*	Enhanced growth phenotype in the *mns3 rsw2-1*^b^ double mutant	Liebminger et al. (2009)
MNS1 MNS2 (MAN1A1) (MAN1A2) (MAN1C1)	At1g51590 At3g21160	*mns1 mns2* *mns1 mns2 mns3*	Double mutant displays a conditional root phenotype; severe growth defect in the *mns1 mns2 rsw2-1* triple mutant Triple mutant displays root and shoot growth phenotype	Liebminger et al. (2009) Liebminger et al. (2010) Liebminger et al. (2009)
GnTI (MGAT1) GMII (MAN2A1) GnTII (MGAT2) XYLT FUT11 FUT12 GALT1 FUT13 *O. sativa* GCSI (MOGS) GnTI (MGAT1) XYLT	At4g38240 At5g14950 At2g05320 At5g55500 At3g19280 At1g49710 At1g26810 At1g71990 Os01g69210 Os02g58590 Os08g0503800	*cgl1 C5* *gntI/cgl1-T* *hgl1-1* *gntII* *xylt* *fut11 fut12* *fut11 fut12 xylt* *hgl1 fut11 fut12* *galt1-1* *fut13* *osmogs* *gnt1* *rcn11*	Increased sensitivity towards salt stress, enhanced growth phenotype in the *gntI rsw2-1* double mutant Increased sensitivity towards salt stress, enhanced root growth phenotype in the *hgl1 rsw2-1* double mutant No described phenotype No described phenotype No described growth phenotype, root growth phenotype in the *fut11 fut12 rsw2-1* triple mutant Increased sensitivity towards salt stress Increased salt sensitivity compared with *hgl1* No described phenotype No described phenotype Hypomorphic allele, defect in root cell division and root elongation Severe growth defects, early lethality, no reproduction Affects vegetative growth under low-temperature conditions	Kang et al. (2008) Kang et al. (2008) Rips et al. (2014) Yoo et al. (2015) Rips et al. (2014) Strasser et al. (2004) Strasser et al. (2004) Rips et al. (2014) Kang et al. (2008) Kaulfürst-Soboll et al. (2011) Strasser, Bondili, Vavra, et al. 2007 Strasser et al. (2008) unpublished Wang et al. (2014) Fanata et al. (2013) Takano et al. (2015)

^a^For conserved enzymes, abbreviations according to the Human Genome Nomenclature Committee are given in parentheses.

^b^*rsw2-1* is a partial loss-of-function mutant of *KORRIGAN1*.

The next *N*-glycan processing step is the removal of a single α1,2-linked Man from the middle branch (B-branch, Figure 2A) of the oligosaccharide. The class I α-mannosidase MNS3 displays this typical ER-α-mannosidase I activity in *A. thaliana* (Liebminger et al. 2009). MNS3-deficient plants produce considerable amounts of incompletely processed *N*-glycans and minor amounts of complex *N*-glycans. The majority of these *N*-glycans carry the terminal α1,2-linked Man on the B-branch providing further evidence that MNS3 activity is responsible for this trimming reaction (Liebminger et al. 2009). *N*-Glycan analysis and enzymatic assays have demonstrated that *A. thaliana* β1,2-*N*-acetylglucosaminyltransferase I (GnTI) is able to transfer a single GlcNAc residue to this uncommon acceptor substrate. Together with further processing by other Golgi-resident enzymes, a number of aberrant complex *N*-glycans are generated in the *mns3* mutant which are well tolerated and do not affect plant development. However, genetic interaction analysis shows a strong synergistic effect between the MNS3 loss-of-function mutant and *rsw2-1* a weak allele of the *KORRIGAN1* gene (Liebminger et al. 2009). A similar additive effect was observed when mannosidase trimming was blocked in the partial loss-of-function mutant *cob-1*, which has a mutation in the gene coding for the *A. thaliana* glycoprotein COBRA (Liebminger et al. 2009). A weaker, but still discernible phenotype enhancement was also detected for a null mutant of the cellulose synthase catalytic subunit 6, which is very likely not N-glycosylated. Collectively, current data suggest that MNS3 and other α-mannosidases are required for the *N*-glycan processing of one or more glycoproteins involved in cellulose/cell wall biosynthesis.

Strikingly and in sharp contrast to the corresponding yeast ortholog, MNS3 does not display any ER location and fluorescently labelled MNS3 is found in puncta resembling Golgi-like structures (Liebminger et al. 2009). *N*-Glycans from ER-resident proteins display substantial amounts of Man_8_GlcNAc_2_ structures (Pagny et al. 2000; Strasser et al. 2008), indicating that these proteins have been processed by MNS3, the only known plant α-mannosidase that can efficiently hydrolyze this Man residue in plants. How can this puzzle be explained? At steady state, MNS3 may have a dual localization in plants with the majority of MNS3 in Golgi-like structures and only a very minor fraction concentrated in the ER where it trims Man residues from the B-branch of glycoproteins. Alternatively, MNS3 may be either completely absent from the ER and concentrated in the Golgi or at the ER-Golgi interface. In this scenario, substrate glycoproteins including ER-resident proteins cycle from the ER to the MNS3 compartment for processing and back to the ER. Although initially found in the ER (Roth et al. 1990; Gonzalez et al. 1999), mammalian ER-α-mannosidase I has been proposed to reside in the Golgi (Pan et al. 2011; Iannotti et al. 2014) and/or in so-called ER-derived quality control vesicles (Benyair et al. 2015). In plants, such vesicles or a dedicated quality control compartment have not been identified. Advanced cell biological studies including high-resolution imaging technologies are required to determine the precise MNS3 location in plant cells. Hopefully, these insights will also shed more light on the functional relevance of this uncommon subcellular compartmentation in plants.

Apart from *N*-glycan processing, yeast and human ER-α-mannosidase I play also a critical role for the disposal of terminally misfolded glycoproteins by the ER-associated degradation (ERAD) pathway. In *S. cerevisiae* the trimming by Mns1p is required for further processing by Htm1p. This stepwise Man removal process leads to the formation of an oligomannosidic glycan with an exposed α1,6-linked Man residue that serves as the glycan signal for degradation of aberrant glycoproteins (Clerc et al. 2009). Plants have a similar ERAD pathway (Liu and Li 2014), but genetic evidence indicates that *A. thaliana* MNS3 is dispensable for the disposal of membrane-anchored or luminal glycoprotein ERAD substrates (Hüttner, Veit, Vavra, Schoberer, Dicker, et al. 2014; Hüttner, Veit, Vavra, Schoberer, Liebminger, et al. 2014). While the *N*-glycan processing by MNS3 and Golgi-α-mannosidases is apparently not a prerequisite for ERAD of misfolded glycoproteins in *A. thaliana*, the activity of two other class I α-mannosidases, MNS4 and MNS5 is crucial to produce the glycan degradation determinant (Hüttner, Veit, Vavra, Schoberer, Liebminger, et al. 2014). The generated *N*-glycan is subsequently recognized by the Man-6-phosphate receptor homology domain-containing lectin OS9 and other components of the ERAD pathway such as HRD3/SEL1L (Su et al. 2011, 2012; Hüttner et al. 2012). Although the molecular mechanisms for terminal α1,6-linked Man formation and recognition are in principle conserved between eukaryotic species, the *N*-glycans of plant ERAD substrates display considerable amounts of mono-glucosylated structures (Hüttner, Veit, Vavra, Schoberer, Dicker, et al. 2014; Hüttner, Veit, Vavra, Schoberer, Liebminger, et al. 2014) (Figure 3). The significance of this difference is unclear, but it is plausible that the CNX/calreticulin cycle and the ERAD machinery interact very closely in plants. Targeting of misfolded glycoproteins for disposal might include a so far uncharacterized step in the quality control and degradation pathway. Whether such an alternative scenario is specific for plants or a more common feature of glycoprotein ERAD remains to be shown. Removal of the Man from the A-branch (Figure 2A) has been proposed to be an important step during quality control and ERAD of misfolded glycoproteins in mammals as it prevents re-glucosylation by UGGT (Avezov et al. 2008). However, a recent study reports that mono-glucosylated proteins can be sent for degradation in mammalian cells similar to the observations for plant ERAD substrates (Tannous et al. 2015). Another open question related to glycan-dependent ERAD is the recognition process of terminally misfolded glycoproteins and the separation from proteins that are still capable of proper folding. Here, MNS4 and MNS5 may be involved, for instance, together with protein disulfide isomerases the α-mannosidases may act as sensors to discriminate between immature folding intermediates and terminally misfolded glycoproteins (Gauss et al. 2011).

Apart from degradation of misfolded glycoproteins, the physiological role of glycan-dependent ERAD is currently unknown in plants and no endogenous glycoprotein or biosynthetic pathway regulated by glycan-dependent ERAD has been discovered. Plants lacking MNS4/MNS5 or other ERAD components do not display any obvious phenotype under normal growth conditions, but are markedly more sensitive to salt stress and ER stress causing agents like tunicamycin (Liu et al. 2011; Hüttner et al. 2012; Hüttner, Veit, Vavra, Schoberer, Liebminger, et al. 2014; Su et al. 2012).

## Complex *N*-glycan formation and function in plants

**Fig. 4.**
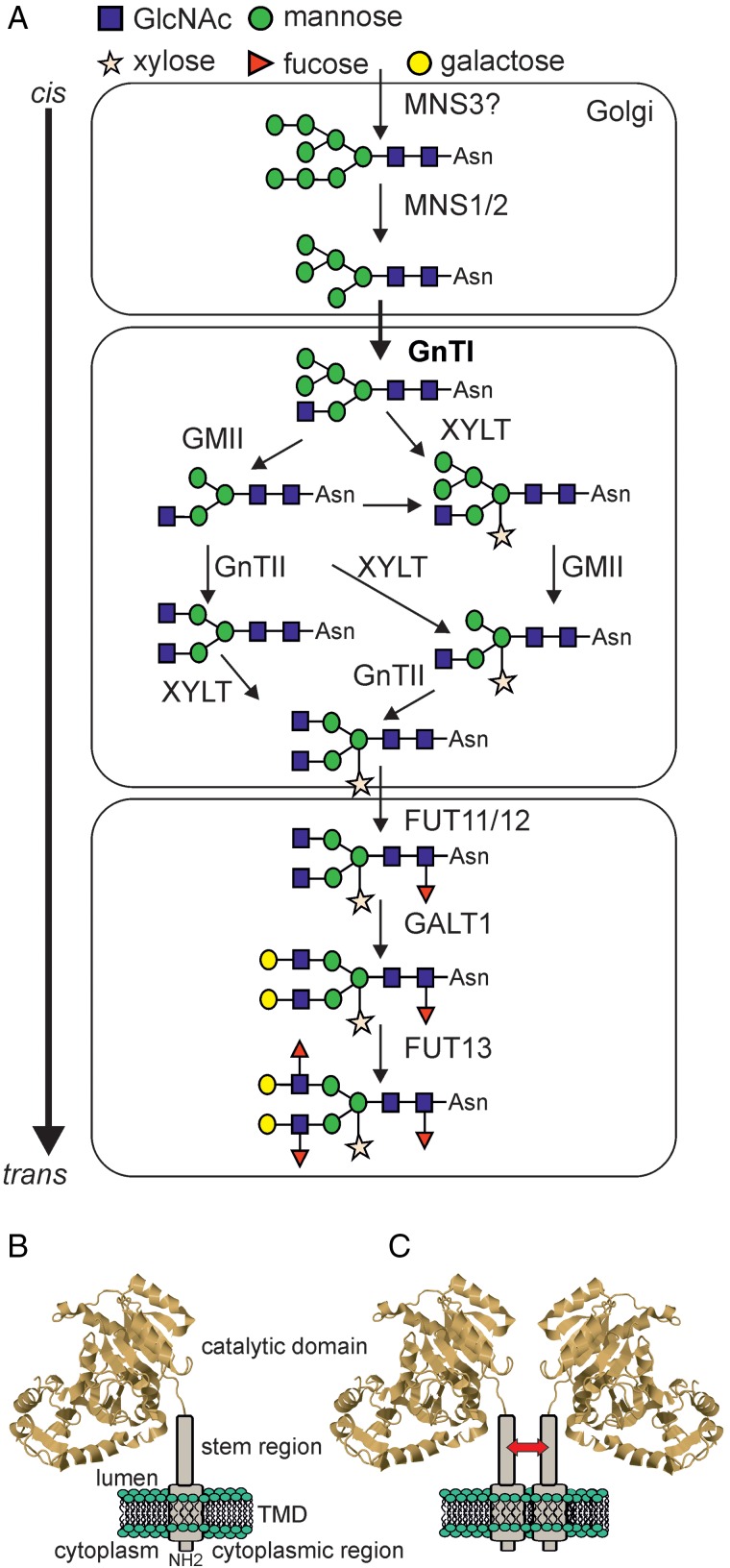
(**A**) Complex *N*-glycan formation in the Golgi apparatus. Terminal Man residues are removed by class I α-mannosidases (MNS1-3). Man_5_GlcNAc_2_ is used by β1,2-*N*-acetylglucosaminyltransferase I (GnTI, highlighted in bold) to initiate complex *N*-glycan formation. The different *N*-glycan processing enzymes required for the maturation of complex *N*-glycans in the Golgi are shown. Note, possible cargo transport processes mediated by cisternal maturation, vesicular transport or tubular connections are not indicated. Dependent on the mode of cargo transport there are differences in localization and retention of glycosylation enzymes. (**B**) Schematic illustration of a Golgi-resident *N*-glycan processing enzyme. The structure of the catalytic domain from rabbit GnTI (Unligil et al. 2000) is illustrated with N-terminal regions representing the short cytoplasmic tail, the single transmembrane domain and the stem region. (**C**) Golgi-resident glycosyltransferases may form homomeric or heteromeric complexes that could be important for concentration of the enzymes in different Golgi cisternae and/or for the modulation of their enzymatic activities. The arrow indicates that tobacco GnTI interacts through its stem region (Schoberer et al. 2014). This figure is available in black and white in print and in color at *Glycobiology* online.

The first *N*-glycan processing step in the Golgi is performed by Golgi α1,2-mannosidase I, which removes three Man residues from Man_8_GlcNAc_2_ to produce Man_5_GlcNAc_2_, the substrate for the subsequent formation of hybrid and complex *N*-glycans. The two *A. thaliana* Golgi α-mannosidases, MNS1 and MNS2, are probably the result of a recent genome duplication event in *A. thaliana* and have redundant function (Liebminger et al. 2009). Like the soybean ortholog they are located in early Golgi cisternae and display the characteristic enzymatic features of class I α-mannosidases like sensitivity to class I α-mannosidase inhibitors kifunensine and 1-deoxymannojirimycin (Nebenführ et al. 1999; Liebminger et al. 2009; Kajiura, Koiwa, et al. 2010; (Figure 4A).

The formation of complex and hybrid *N*-glycans is initiated by GnTI, which transfers a single GlcNAc residue to the α1,3-linked Man of the Man_5_GlcNAc_2_ acceptor substrate to create GlcNAcMan_5_GlcNAc_2_ (von Schaewen et al. 1993; Strasser et al. 1999). Similar to animals, the GlcNAc transferred by GnTI is absolutely required for all subsequent *N*-glycan processing steps in the Golgi. Due to its central role in the pathway numerous studies have focused on the characterization of its biochemical characteristics, subcellular localization and physiological function. * Arabidopsis thaliana* mutants lacking GnTI activity were initially isolated from a pool of EMS mutagenized seeds (von Schaewen et al. 1993). These *complex glycan 1* (*cgl1*) mutants do not produce complex *N*-glycans, when cultivated under normal growth conditions, but one of the characterized *cgl1* alleles can be suppressed in plants with reduced N-glycosylation efficiency (Frank et al. 2008; Farid et al. 2011). More recently a T-DNA insertional mutant in the GnTI gene has been described (*cgl1-T* or *gntI*) (Frank et al. 2008). Apart from slight differences during development (e.g. longer flowering time) (von Schaewen et al. 1993; Boyes et al. 2001) these GnTI-deficient *A. thaliana* do not display any phenotype under normal growth conditions. However, the *cgl1* plants display hypersensitivity towards salt stress (Kang et al. 2008), which is similar to the salt sensitivity of the OST mutant *stt3a-2*. In summary, the findings for *A. thaliana* GnTI-deficient plants suggest that complex *N*-glycans are not essential for their survival, which is in contrast to the embryo lethal phenotype of GnTI knockout mice (Ioffe and Stanley 1994; Metzler et al. 1994). Recently, a GnTI T-DNA insertional mutant lacking complex *N*-glycans has been characterized from *Oryza sativa*. The rice *gnt1* mutant displays severe defects in organ development that are accompanied by problems in reproduction (Fanata et al. 2013). Although the authors have not succeeded in complementation of the *gnt1* line, it is very likely that GnTI activity and complex *N*-glycans are essential for rice plants. Based on this new finding, it is plausible that other plant species will also display severe developmental defects when complex *N*-glycan formation is completely blocked.

Immediately after GnTI action, alternative processing reactions can take place in plants because GlcNAcMan_5_GlcNAc_2_ serves as substrate for at least three different *N*-glycan processing enzymes (Bencúr et al. 2005) (Figure 4A). One possible subsequent step in the plant *N*-glycan processing pathway is the cleavage of two Man residues by Golgi-α-mannosidase II (GMII). Like in other higher eukaryotes, the Man removal is followed by β1,2-*N*-acetylglucosaminyltransferase II (GnTII)-catalyzed transfer of another GlcNAc residue to the α1,6-linked Man. Alternatively, the hybrid GlcNAcMan_5_GlcNAc_2_ glycan can be modified in the *medial*-Golgi by β1,2-xylosyltransferase (XYLT) or *medial*- to *trans*-Golgi by core α1,3-fucosyltransferase. Both enzymes are ubiquitous in plants, but not present in mammals. These alternative processing routes have been postulated based on the analysis of the substrate specificity of recombinant enzymes and have been supported by the analysis of *N*-glycan structures in corresponding knockout plants (Strasser et al. 2006). In agreement with data for GnTI-deficient *A. thaliana*, the characterized GMII and GnTII knockout plants do not display any obvious phenotype under normal growth conditions. However, GMII-deficient *A. thaliana* display a conditional phenotype under salt stress (Kang et al. 2008).

*Arabidopsis thaliana* contains a single XYLT enzyme capable of transferring Xyl residues to various *N*-glycan acceptor substrates in vitro and in vivo (Strasser et al. 2004, 2006; Bencúr et al. 2005). As mentioned before, XYLT can compete for the same substrates with core α1,3-fucosyltransferase. Two core α1,3-fucosyltransferases, FUT11 and FUT12, have been identified in *A. thaliana* and both can modify *N*-glycans in vivo in a redundant manner (Wilson, Rendić, et al. 2001; Strasser et al. 2004). The presence of two core α1,3-fucosyltransferase copies in the *A. thaliana* genome is most likely the result of a gene duplication event, which is corroborated by the fact that only a single core α1,3-fucosyltransferase gene is present in plant species like rice (Léonard et al. 2004). While XYLT and FUT11/FUT12 can utilize the same acceptor *N*-glycan, preceding core α1,3-fucosylation interferes with in vitro xylosylation of *N*-glycan substrates (Bencúr et al. 2005). Interestingly, *N*-glycans lacking core Fuc are increased in XYLT-deficient *A. thaliana*, indicating that attachment of β1,2-Xyl enhances the rate of Fuc transfer (Strasser et al. 2004, 2008; Kaulfürst-Soboll et al. 2011). The subcellular localization of core α1,3-fucosyltransferases has not been reported, but it has been suggested that core fucosylation occurs mainly in the *medial*- and *trans*-Golgi (Lerouge et al. 1998). Interestingly, neither the single *A. thaliana* (*xylt*, *fut11*, *fut12*), nor double (*fut11 fut12*), nor triple (*xylt fut11 fut12*) knockout lines display any obvious phenotype under normal growth conditions indicating that β1,2-Xyl and core α1,3-Fuc residues are dispensable for plant development. The absence of a visible phenotype in stable mutant lines is consistent with *N. benthamiana* plants with strongly downregulated expression of XYLT and core α1,3-fucosyltransferases (Strasser et al. 2008). Under salt stress conditions no or only a very weak root growth inhibition has been detected for *A. thaliana xylt* and *fut11 fut12* but a stronger phenotype has been described for the *xylt fut11 fut12* triple mutant (Kang et al. 2008).

The last known step in the *N*-glycan processing pathway in plants is the generation of Lewis A-containing structures. Two enzymes are required for the synthesis of the Lewis A epitope on complex plant *N*-glycans (Lerouge et al. 1998; Strasser, Bondili, Vavra, et al. 2007). First, a β1,3-galactosyltransferase (GALT1) transfers Gal in β1,3-linkage to terminal GlcNAc residues, resulting in the synthesis of type 1 chain structures (Galβ1–3GlcNAc). In the second step, α1,4-fucosyltransferase (FUT13) transfers Fuc in α1,4-linkage to the GlcNAc of the type 1 chain to complete the synthesis of the Lewis A structure. Although the *A. thaliana* CAZy GT-31 family consists of 20 putative β1,3-galactosyltransferases, only GALT1 appears to modify *N*-glycans (Strasser, Bondili, Vavra, et al. 2007). *Arabidopsis thaliana* GALT1 is a Golgi-resident type II membrane protein with an uncommon protein architecture. GALT1 contains a putative galactoside binding lectin-domain between the N-terminal targeting/membrane anchoring region and the catalytic domain (Strasser, Bondili, Vavra, et al. 2007). A similar lectin-like domain is not found in mammalian β1,3-galactosyltransferases (Hennet 2002). This characteristic protein domain is also present in five other *A. thaliana* proteins, which belong to CAZy GT-31 family and display 40–73% identity to GALT1 at the amino acid sequence level (Strasser, Bondili, Vavra, et al. 2007; Qu et al. 2008). Members of this lectin-domain-containing sub-family have been recently proposed to initiate arabinogalactan biosynthesis by transfer of Gal to hydroxyprolines on arabinogalactan proteins (Basu et al. 2013, 2015).

GALT1-deficient *A. thaliana* lack the Lewis A epitope on *N*-glycans, but display no growth or developmental defects (Strasser, Bondili, Vavra, et al. 2007). The same applies for a GALT1 overexpression line that produces increased amounts of the Lewis A epitopes on glycoproteins in different *A. thaliana* organs. A T-DNA insertional mutation in the *FUT13* gene leads to the loss of the Lewis A epitope without any effect on plant growth or development (Strasser et al. 2008). The Lewis A-containing glycans are expressed in an organ-specific manner in *A. thaliana* with high amounts in stems and siliques and virtually none in leaves (Fitchette et al. 1999; Wilson, Zeleny, et al. 2001; Strasser, Bondili, Vavra, et al. 2007). Moreover, the epitope is not found on vacuolar proteins, but enriched at the plasma membrane and extracellular glycoproteins. *Arabidopsis thaliana* glycoproteins carrying the epitope have not been characterized. A glycoproteomics study from etiolated *A. thaliana* hypocotyls identified a single glycoprotein with a mono-antennary Lewis A-type *N*-glycan at one of its N-glycosylation sites (Zhang et al. 2011). The function of this cell wall glycoprotein with homology to blue copper binding proteins has not been studied and the biological relevance of Lewis A structures on plant complex *N*-glycans is still obscure.

Apart from the formation of the Lewis A structures, no additional elongation or modification of terminal GlcNAc residues has been identified in plants. As a consequence, plants lack complex *N*-glycans with sialic acid, core α1,6-linked Fuc, β1,4-galactoslyation or branching of *N*-glycans. However, plants are able to efficiently carry out these complex *N*-glycan maturation steps when the missing enzymes and pathways are transiently or stably heterologously expressed *in planta*. Numerous glyco-engineering approaches have demonstrated that plants tolerate these modifications very well and are able to produce defined mammalian-type *N*-glycan structures in substantial amounts (Strasser et al. 2014).

Are there any post-Golgi modifications of *N*-glycans? Earlier studies with common beans have shown that terminal GlcNAc residues are removed from Golgi-modified glycoproteins in the vacuole giving rise to the formation of paucimannosidic *N*-glycans (Vitale and Chrispeels 1984; Lerouge et al. 1998). More recent data extend this view and indicate that post-Golgi processing of *N*-glycans takes place in the vacuole as well as at the plasma membrane (Strasser, Bondili, Schoberer, et al. 2007; Liebminger et al. 2011; Castilho et al. 2014). In *A. thaliana*, the specific cleavage of terminal GlcNAc residues from vacuolar glycoproteins is carried out by β-*N*-acetylhexosaminidase 1 (HEXO1). In contrast, β-*N*-acetylhexosaminidase 3 (HEXO3) has been located to the plasma membrane where it trims GlcNAc residues from secreted glycoproteins (Liebminger et al. 2011). While the biological functions of HEXO1 and HEXO3 are unknown, it has been shown for other plant species that β-*N*-acetylhexosaminidases are important factors for the control of fruit ripening (Meli et al. 2010).

## Golgi organization of plant *N*-glycan processing enzymes

An open question in plant glycobiology is the spatial organization of glycosyltransferases and glycosidases in the secretory pathway, especially in the Golgi, the site of complex *N*-glycan formation and cell wall polysaccharide synthesis. In particular, how the non-uniform distribution of Golgi-resident enzymes is achieved and maintained during constant trafficking of cargo glycoproteins is largely unknown (Schoberer and Strasser 2011). The distribution of *N*-glycan processing enzymes in a *cis/medial-*to-*trans* fashion along the different Golgi cisternae make them valuable tools to investigate the spatial organization and underlying mechanisms (Rabouille et al. 1995). Tobacco GnTI, the central enzyme for complex *N*-glycan maturation is a type II membrane protein. The signal for Golgi targeting and retention of tobacco GnTI is present in the so-called cytoplasmic tail, transmembrane domain and stem region (Schoberer et al. 2009, 2014) (Figure 4B). Remarkably, the basic principles of Golgi targeting and retention appear conserved as plant and mammalian GnTI can complement each other (Gomez and Chrispeels 1994; Bakker et al. 1999). Moreover, the N-terminal targeting region from the *trans*-Golgi-resident rat α2,6-sialyltransferase is the most widely used *trans*-Golgi marker in plants (Boevink et al. 1998). However, glyco-engineering approaches have also shown that human β1,4-galactosyltransferase 1 (B4GALT1) is targeted to a different Golgi subcompartment in plants and interferes with biantennary complex *N*-glycan formation leading to increased *N*-glycan heterogeneity (Bakker et al. 2001; Strasser et al. 2009). Such subtle differences in Golgi targeting and retention may be attributed to distinct mechanisms that control Golgi organization of glycosyltransferases (Tu and Banfield 2010). In yeast and mammalian cells, for instance, a sorting mechanism based on recognition of sequence motifs within the cytoplasmic tail of glycosyltransferases has been revealed (Schmitz et al. 2008; Tu et al. 2008; Ali et al. 2012; Pereira et al. 2014). In plants, the corresponding binding motif as well as a homolog of the protein sorting determinant (yeast Vps74p/mammalian GOLPH3) has not been found (Schoberer and Strasser 2011), suggesting that plants may use a different localization mechanism. Another largely unexplored factor is the role of the lipid composition and membrane environment in the plant Golgi. In mammalian cells, it has been proposed that changes in lipid composition and specialized membrane microdomains lead to partitioning and sorting of membrane-anchored proteins (Lippincott-Schwartz and Phair 2010).

FRET-FLIM and co-immunoprecipitation experiments have demonstrated that early Golgi *N*-glycan processing enzymes like MNS1 and GnTI can form homomeric complexes and these sequential acting enzymes interact with each other in vivo (Schoberer et al. 2013) (Figure 4C). While the biological significance of the identified protein complex formation is unclear for plant *N*-glycan processing enzymes, it appears that complex formation in the Golgi is a common feature in different glycosylation pathways and organisms (Atmodjo et al. 2011; Chou et al. 2015; Kellokumpu et al. 2016). The pectin biosynthetic galacturonosyltransferase GAUT1 is retained in the Golgi by complex formation with GAUT7 (Atmodjo et al. 2011). The association between GAUT1 and GAUT7 proteins is mediated by covalent disulfide bonds and non-covalent interactions. A specific protein–protein interaction may fine-tune enzyme activity (Hassinen et al. 2011) or alternatively, provide a common mechanism for Golgi retention or recycling of enzymes that act together in the same biosynthetic pathway (Nilsson et al. 1993). On the other hand, recent data from complementation studies of GnTI-deficient plants with different chimeric GnTI variants suggests that efficient complex *N*-glycan formation in *A. thaliana* is possible without homo- or hetero-dimer formation (Schoberer et al. 2014). Clearly, many fundamental questions related to the Golgi organization of *N*-glycan processing enzymes are unsolved in plants and more emphasis should be given to cell biological aspects of protein glycosylation which are essential to understand the complex regulation of glycan modifications in all higher eukaryotes.

## Are complex *N*-glycans required for the function of the plant glycoprotein KORRIGAN1?

As mentioned before, there is not much known about the role of complex *N*-glycans for distinct plant glycoproteins. The *A. thaliana* endo-1,4-β-d-glucanase KORRIGAN1 (KOR1) is a membrane-anchored glycoprotein involved in cellulose biosynthesis (Nicol et al. 1998). The KOR1 partial loss-of-function mutant *rsw2-1* displays a temperature-sensitive root growth phenotype (Lane et al. 2001). Interestingly, GnTI-deficiency strongly enhances the *rsw2-1* root growth phenotype even at the permissive temperature (Kang et al. 2008). A similar additive phenotype with severe developmental defects has been observed for *mns3 rsw2-1* and *mns1 mns2 rsw2-1* mutants that harbor mainly oligomannosidic *N*-glycans (Liebminger et al. 2009, 2010). These findings suggest that one or several of the eight *N*-glycans from KOR1 require complex *N*-glycans. Mass spectrometry and immunoblots has revealed that KOR1 carries oligomannosidic as well as complex *N*-glycans and heterologous expression of KOR1 variants lacking individual N-glycosylation sites in insect cells has demonstrated that N-glycosylation is important for in vitro enzyme activity (Liebminger et al. 2013). However, a recombinant KOR1 variant with essentially oligomannosidic *N*-glycans displays a comparable in vitro activity like KOR1 with complex *N*-glycans. In another study, it has been shown that KOR1 lacking all eight N-glycosylation sites is still able to partially complement the root growth phenotype of *gntI rsw2-1* plants (Rips et al. 2014). In summary, both studies independently conclude that complex *N*-glycans are very likely not directly required for KOR1 function. Thus, an unknown glycoprotein with complex *N*-glycans and involvement in cellulose synthesis appears affected in these plants and causes the observed additive phenotype. Further genetic evidence shows that GMII-deficiency as well as the absence of core Fuc also enhances the *rsw2-1* phenotype (Rips et al. 2014). On the other hand, GnTII-deficiency or the lack of Lewis A epitope formation does not lead to an additional phenotypic effect. Future genetic and biochemical studies should aim to identify this unknown glycoprotein with a functional complex *N*-glycan structure in plants.

## Conclusion and future perspectives

The *N*-glycan processing pathway in plants is quite well understood and all enzymatic steps leading to the formation of the known *N*-glycan structures have been investigated. However, even from the model plant *A. thaliana* a comprehensive *N*-glycan profiling of different organs and cell types has not been reported and information on *N*-glycan maturation steps in different plant species is incomplete. Intriguingly, studies of the *A. thaliana* Golgi proteome revealed numerous Golgi-resident glycosyltransferases of unknown function (Nikolovski et al. 2012; Parsons et al. 2012). While most of them are very likely involved in the biosynthesis of different cell wall polysaccharides or complex O-glycosylated proteins like arabinogalactan proteins it cannot be excluded that some of these enzymes generate rare modifications on *N*-glycans. In comparison to mammals, our understanding of *N*-glycan function is very limited in plants. Data for *A. thaliana* and for monocots like rice strongly indicate that complex *N*-glycans are crucial for correct growth under stress conditions. The recent identification of rice XYLT-deficient plants with significant growth defects at low temperature is one example (Takano et al. 2015). The complete sequencing of different plant genomes and the availability of straightforward genome editing technologies will pave the way for efficient modifications of *N*-glycan processing reactions in many plant species within a reasonable time frame (Quétier 2016). Likewise, these editing tools can be applied to introduce mutations into the genome that remove N-glycosylation sites from individual glycoproteins in order to investigate the biological role of distinct *N*-glycans under different environmental conditions. As a consequence of these revolutionary developments novel functions of plant *N*-glycans will be revealed soon.

## Funding

This work was supported by the Austria Science Fund (FWF) (grant number P23906-B20). Funding to pay the Open Access publication charges for this article was provided by the Austrian Sience Fund (FWF) (grant number P23906-B20).

## Conflict of interest statement

None declared.

## Abbreviations

ALG, asparagine-linked glycosylation; EFR, EF-TU RECEPTOR; EMS, ethyl methanesulfonate; ER, endoplasmic reticulum; ERAD, ER-associated degradation; FLIM, fluorescence lifetime imaging; FRET, Förster resonance energy transfer; FUT, fucosyltransferase; GnTI, β1,2-*N*-acetylglucosaminyltransferase; GCS, α-glucosidase; KOR1, KORRIGAN1; MNS, α-mannosidase; OST, oligosaccharyltransferase; UGGT, UDP-Glc glycoprotein glucosyltransferase; XYLT, β1,2-xylosyltransferase.
